# Toward a sustainable growth path in Arab economies: an extension of classical growth model

**DOI:** 10.1186/s40854-022-00426-6

**Published:** 2023-01-12

**Authors:** Amjad Taha, Mucahit Aydin, Taiwo Temitope Lasisi, Festus Victor Bekun, Narayan Sethi

**Affiliations:** 1 Department of Banking and Finance, Eastern Mediterranean University, North Cyprus Via Mersin 10, Famagusta, Turkey; 2 Department of Econometrics, Faculty of Political Sciences, Sakarya University, Esentepe Campus, Serdivan, Sakarya Turkey; 3 Department of Recreology and Tourism, Faculty of Informatics and Management, University of Hradec Králové, Hradec Kralove, Czech Republic; 4 Faculty of Economics Administrative and Social Sciences, Istanbul Gelisim University, Istanbul, Turkey; 5 Adnan Kassar School of Business, Lebanese American University, Beirut, Lebanon; 6 Department of Humanities and Social Sciences, National Institute of Technology (NIT) Rourkela, Rourkela, 769008 Odisha India

**Keywords:** Arab economies, Classical growth model, Panel econometrics, SDG, Savings-investment

## Abstract

**Background/Objectives:**

Many economies are on the trajectory of alternative growth drivers other than conventional capital and labor. Access to credit facilities is a pertinent indicator of economic growth. In line with the United Nations Sustainable Development Goals (UNSDGs-8) agenda, the national goal for sustainable development for most economies and Arab economies is no exception. Therefore, the current study adopts a traditional growth model by exploring the relationship between gross domestic product (GDP) per capita, credit for private sectors, ratio of exports, real GDP, and per labor force participants for selected Arab economies annually from 2001 to 2020.

**Research design:**

This study leverages the Fourier Kwiatkowski–Phillips–Schmidt–Shin (KPSS) unit root test and second-generation panel econometrics as estimation techniques, such as Westerlund and Edgerton panel cointegration test, and the use of two estimators, namely the augmented mean group (AMG) and common correlated error mean group (CCEMG), to obtain robust results.

**Findings:**

Empirical findings from Westerlund and Edgerton panel cointegration tests validate the long-run equilibrium relationship among the outlined variables. Further empirical results indicate that the share of exports is negatively significant with economic growth in countries such as Kuwait, Lebanon, Tunisia, and Jordan. Additionally, savings and labor force participation have a positive relationship with economic growth in individual countries such as Algeria and Bahrain. As per the panel, there is no significant relationship between labor force participation and economic growth. This indicates that the skilled labor force enhanced economic growth.

**Conclusions:**

These findings come with inherent far-reaching policy suggestions for economies and panels. Further details on country-specific policy actions are presented in the concluding section.

**Graphical Abstract:**

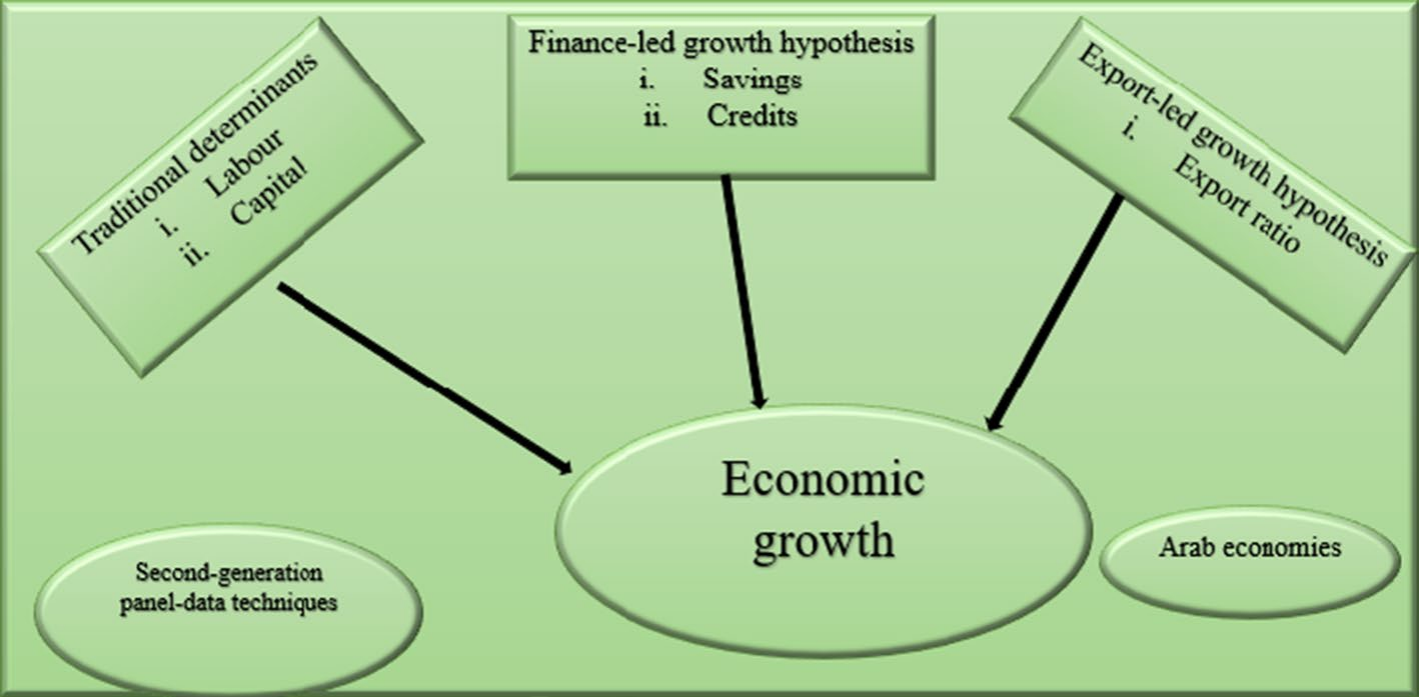

## Introduction

Traditional growth theories, such as endogenous and exogenous economic growth theories, were developed to understand and explain the sources of economic growth. While several factors have been propagated as sources of growth (Solow 1956), technological progress, workforce, and investments are critical sources of economic growth viability. Nevertheless, the efficient use and size of economic variables affect the magnitude of a country’s economic growth, particularly after the pandemic (Wang and Zhang 2021; Wang and Su 2020). In other words, growth is a function of input, and a country can only grow in proportion to its input level. Comparative economic well-being and performance are ascertained through measures of economic growth, which is a function of technological changes and capital accumulation. Population growth and savings rates (Solow 1956), as well as trade and access to credit (Osipian 2007), are determinants of capital accumulation, which contributes to economic growth.

Capital accumulation also occurs through exports (Śledziewska and Akhvlediani 2017; Hou and Karayalcin 2019), which affect economic growth. Exporting goods and services, particularly in developing nations, can further improve the nations’ domestic economies, as exports are a component of economic growth; therefore, export growth leads to economic growth (Islam and Haque 2018). Additionally, exports indirectly influence economic growth by accelerating technological advancement, improving productivity, and enhancing the efficiency of economic resource allocation. Any population’s labor supply is determined by its size (grouped by age and sex) and the labor force participation for each group (Abraham and Kearney 2020). The labor force is considered an asset for a nation and is believed to be a common occurrence in many developing countries (Haque et al. 2019). According to Girón and Kazemikhasragh (2021), gender opportunities and outcomes are presented in several dimensions, such as access to formal employment, occupation, and education. Sasongko et al. (2020) and Baerlocher et al. (2021) have attempted to verify or reject arguments regarding the impact of labor force participation on economic growth. Findings from previous studies suggest that labor force participation over a long period is a function of the social, economic, and demographic trends of a nation. In particular, in the Arab world, there is a low level of female labor participation, more because of religion than culture (Korotayev et al. 2015).

Appertaining to economic stability, savings are the most essential source of funds; however, the saving-investment balance is also critical for attaining sustainable economic growth (Joshi et al. 2019). Owing to the lack of capital and savings, private sectors and households in emerging economies constantly incur debt, while public sectors expand by receiving a larger percentage of national income (Hungwe and Odhiambo 2019). In addition, savings and investments have been highly correlated since the mid-twentieth century (Segura 2019). For instance, Keynes (1931, 1936) cautioned against drawing incorrect conclusions about the relationship between investment and saving because it is possible to invest without saving or saving without investing. Some of these studies suggest that spending, rather than saving, positively affects individual income and savings, while others suggest that increasing savings does not increase economic growth. However, it is commonly believed that a lack of savings poses a problem for sustainable growth (Alkin 2016). According to Azar et al. (2018a, b), the Arab national savings trailed below emerging economies, coming up at 24.4% of gross domestic product (GDP) compared to middle-income countries’ 31.5% and the growing Asian economies’ 36.2%. Based on this performance, it is intriguing to consider how private savings, compared to politically motivated government savings, contributed to these results. More importantly, what prospective contributions can they make to enhance the Arab economy and ensure sustainability?

The Middle East and North Africa (MENA) region has enormous natural and human resources, contributes a substantial share of global petroleum production and exports, and has an average acceptable quality of life (World Bank 2021). Within this broad classification, countries differ significantly in terms of resources, economic and geographical sizes, populations, and living conditions. More recent issues have emerged in the Arab region because of the coronavirus disease (COVID-19) outbreak, volatility of oil prices, and many economic structural challenges. Considering the expanding gaps, Arab countries have similar labor market characteristics and face similar issues. High unemployment rates, particularly among youth; low labor force participation, particularly among women; significant but decreasing proportions of public sector employment; and a high frequency of unofficial employment are issues faced by the labor market (United Nations Development Programme 2020a). The labor force participation in the region was 45.55%, a 2.38% decline from the previous year, and much lower than the global average of 58.61% (World Bank 2022b), which is mainly due to women’s low participation rate of 19.63%.

The relevance of financial access to growth in the private sector cannot be overemphasized. A small amount of bank loans goes to small- and medium-sized enterprises (SMEs) in the oil-exporting Arab nation, with a small percentage of organizations with loans or credit lines from financial institutions. The domestic credit to the private sector in the Arab region experienced an upward trend between 2012 and 2016 but a 3.12% decline in 2017. In Yemen, Iraq, and Algeria, for instance, while credit growth is substantial, bank lending to the private sector amounts to only 10–30% of non-oil GDP (International Monetary Fund 2016). The current downturn in oil prices is attributed to the drying-up of surplus revenue in many oil-exporting Arab economies (e.g., Algeria and the Gulf Cooperation Council); consequently, there is a concern that liquidity in some of these countries may become extremely limited. The private sector’s access to credit may be stifled because of restricted liquidity conditions, particularly when government borrowing from banks increases, which also puts an extra strain on the financial system (Kou et al. 2021, 2022).

Furthermore, in the last two decades, owing to economic dislocations and political insecurity in the region, they have experienced comparatively low national savings (Azar et al. 2018a, b). Between 2018 and 2020, the region experienced a 35.88% decline in its gross domestic savings compared to the 1.39% global decline and 36.69% decline in the MENA economies. In addition, since 2000, the Arab countries’ total proportion of global exports has doubled, reaching 7% in 2014. In 2015, fuel exports accounted for 73% of the region’s commerce, representing the majority of exports to oil-producing countries. Non-oil exports from the Arab region, excluding fuel, accounted for 2% of global exports in 2013, up from 1% in 2000 (United Nations Development Programme 2020b).

### Why Arab economy?

According to Khalid and Hassan (2022), after sub-Saharan Africa, households in Arab nations have the second lowest rate of growth pass-through in the world. The impact of the COVID-19 pandemic is partially responsible for the weak growth pass-through, which is visible even in several high- and middle-income Arab nations. In addition, the Arab economy is a mixed economy; however, the majority of countries fall into the middle economy category. This can lead the region’s economy to experience the “middle-income trap,” where economies that are fast-expanding stagnate at middle-income levels rather than rising to the level of high-income nations. Furthermore, the sad reality is that a decade after the Arab Spring, the region’s states were in a far worse position than they were in terms of all three dimensions of Bertelsmann Stiftung’s Transformation Index (BTI 2022). Almost all expected benefits of democratic advancement, economic engagement, and social fairness remain unrealized. The region’s most promising signals emerge from a failing state, which is a sign of a dire situation. Since the Bertelsmann Stiftung’s Transformation Index (BTI 2022), the region as a whole has seen a new economic low point, which is comparable to the scenario during the start of the Arab Spring. This downturn even corresponds to an entire point on the BTI’s 10-point scale, making it the greatest downward trend ever observed in the region.

Additionally, the output of the oil and non-oil sectors will be stimulated by a number of supportive factors, which are expected to increase the Gulf Cooperation Council (GCC) growth rate in 2022 to approximately 5.8% (Daily News Egypt 2022), including the effective economic changes implemented to boost levels of economic diversity and encourage foreign and local investment, as well as the beneficial effects of the stimulus packages to aid with the recovery from the COVID-19 pandemic. Under the “Organization of the Petroleum Exporting Countries (OPEC) + ” agreement, the rise in global oil and gas prices is anticipated to benefit other Arab oil-exporting countries, increasing the growth rate of the group in 2022 to 4.6% from 3.30% in the previous year. However, according to the Daily News Egypt (2022), it is anticipated that the growth rate declines in 2023 to 3.9% because of challenges the Arab countries are facing relating to fostering commercial ecosystems and enhancing their attractiveness.

### Contribution of the study

Based on the above synopsis, this study employs a comprehensive approach that considers the interlinkages between economic growth, credit, savings, exports, and labor participation of selected Arab countries toward sustainable economic growth. In particular, this study aimed to answer the following questions:

- What effect does export have on Arab’s economic growth?
- Does financial development augment Arab’s economic growth?
- What effect does labor participation have on Arab’s economic growth?

This article have numerous significant contributions to the literature by responding to these questions. First, the findings from the study sheds light on how Arab economies and other middle-income economies can upturn from the middle-income trap with access to savings and credit, thereby reducing employment. Second, a socioeconomic indicator is considered, which has not been included in related studies, thereby contributing to the literature and providing clear policy directions. Finally, and most importantly, this study used a simple but illustrative one-equation model for the determinant of economic growth, making the research model theoretically based and not ad hoc, and advanced panel data techniques are used.

This study contributes to growth theory and the literature on the determinants of Arabian economic growth.

Given the aforementioned motivation, and despite the plethora of studies concerning the determinants of economic growth, the issue is far from being solved, and the discussion is contentious. The determinants of economic growth may vary by income and regional heterogeneity, as well as by the economic and political structures of the economies considered. Specifically, it is acknowledged that increased savings and credit may not translate into economic growth; improvement in the financial sector does not automatically translate into economic growth. Therefore, studies on the determinants of growth, particularly the finance-growth relationship, are still growing.

Traditional growth theories have been verified and widely accepted, and it has been firmly established that capital, labor, exports, and financial variables are essential drivers of economic growth. However, it is imperative to understand the relative relevance of export- and finance-led hypotheses and the primary growth determinants (labor and capital) for Arab economies. For example, a major attribute of financial development is an increase in savings, credit, and investment. Thus, financial development is tantamount to an increase in the three variables, and it is better to consider them individually rather than in a composite financial index that most recent studies use. Separation is necessary because each variable may have a different effect on the economic growth of the countries considered. Considering the variables individually enables policymakers to determine the exact policy instruments, such as population, trade, or investment policies, –that can be used to spur economic growth. In particular, in Arab economies, it is not established which of the identified variables is crucial for economic growth in the region as a whole and the individual economies. Therefore, it is difficult, if not impossible, for the Arab economies considered in this study to align its policy framework with the findings of previous studies.

Moreover, the methodologies used in previous studies are deficient. Most previous studies have failed to account for cross-sectional dependence in the panel of countries considered. Cross-sectional dependence implies that a shock to a variable in one country will have a spillover effect on the variable in another country. When this is not captured in the analysis, the estimates are invalid and unreliable, resulting to a misleading conclusion regarding the relationship between economic growth and its determinants. The increasing wave of political, financial, and cultural globalization, neighborhood (spatial) effects, and economic integration are the major causes of the cross-sectional correlation among the variables considered. Therefore, as the sample of countries in this study are from the same region, and the countries belong to the same economic union and are open to globalization, data are susceptible to cross-sectional dependence, which are captured by the techniques used in this study.

To bridge this research gap, this study extends the scope of conventional growth models by evaluating the relationship between economic growth, credit, savings, exports, and labor participation in selected Arab economies, which have received little or no attention in the extant economic growth study. Therefore, the contributions of the present study are based on the revisit of the integration of the traditional growth models (finance- and export-led growth hypotheses), the use of a sample of Arab economies (neglected in previous studies), and application of second-generation panel data techniques. This first contribution is that this study provides interesting policy insights on the relevance of the variables considered to the economic growth of the individual Arab economies and the region in general. The channels through which financial development may affect economic growth include savings, credit, and investment. As it has been argued that not all savings are converted to investment, it is imperative to understand the channel through which economic growth occurs in Arab nations. It is important to understand the channels that are viable for the countries considered. Hence, we evaluate the impact of each variable on the economic growth of each country and the entire panel of countries. The second contribution is the use of state-of-the-art techniques of panel data analysis that account for cross-sectional dependence, which is indispensable because of the current wave of globalization. Finally, this study provides viable policy options concerning the economic growth of Arab economies.

The remainder of the paper is structured as follows: a review of the previous literature is presented in Sect. 2. Section 3 discusses the research methodology. Section 4 discusses the findings. Section 5 presents the conclusion/policy recommendations of the study.

## Theoretical background and literature review

The theoretical and empirical literature concerning the drivers of economic growth, particularly financial development, has attracted a considerable number of scholars and policymakers over the years. Other determinants of growth are considered in this study; however, the literature review focuses more on the relationship between financial determinants (savings and credits) and economic growth. This is because the channels through which labor and exports affect economic growth are well established in the theoretical and empirical literature. However, the discussion of finance-led growth remains contentious and continues to expand. However, the literature review in this study is presented thematically, based on the main hypotheses, which have been the center of discourse concerning the determinants of economic growth. First, the traditional determinants of economic growth are considered, followed by finance-led and export-led growth hypotheses.

### Conventional economic growth determinants

The traditional determinants of economic growth (labor, capital, and technology) are mostly considered through numerous theoretical discussions and are confirmed by empirical studies. Neoclassical and endogenous growth theories are distinguished among theories of economic growth. The neoclassical that emerged in the 1950s and was led by the traditional Solow-Swan (Solow 1956) growth model (Solow growth model henceforth) identified labor, capital, and technology as the primary determinants of economic growth. The theory postulates that long-term economic growth depends on the level of capital accumulation, labor (workforce), and technological progress, which are exogenously determined. The main deficiency of Solow’s work is the assumption that technological progress is determined exogenously. In an attempt to remedy the major shortcomings of the Solow model, the endogenous growth model developed in the 1980s posits that the primary determinants of economic growth are labor, capital, and technology, with the assumption that among other things, technological progress is determined endogenously—economic growth is determined by factors shaped within the economy. In short, with different assumptions, both neoclassical and endogenous growth theories show that economic growth depends largely on the level of capital (physical and human capital) accumulation, labor, and technology (Valente 2016). Empirical studies such as those by Lucas (1988), Romer (1994), and, most recently, Eftimoski (2022) identified the presence of unexplained factors in neoclassical growth models and argued that labor abilities, skills, and knowledge (human capital) are the engines of economic growth. Thus, the common conclusion in the study is that labor and capital have a positive relationship with economic growth, leading to the following research hypotheses:

#### Hypothesis 1

There is a positive relationship between labor and economic growth.

#### Hypothesis 2

There is a positive relationship between capital and economic growth.

### Finance-led growth hypothesis

The common feature of neoclassical and endogenous growth models is the savings-investment equality assumption, which implies that savings are equal to investment. However, investors often take loans or issue securities because they do not always have enough money for themselves. Thus, savings-investment equality can only be achieved in a perfectly functioning financial system (market), which is rarely tenable because of information asymmetry and transaction costs (Asanović 2020). This suggests that the effectiveness and efficiency of converting savings into investment is a function of the financial system. Hence, the financial system plays a crucial role in promoting economic growth, a role the financial system plays through the mobilization of savings, allocation of resources to productive investments, facilitation of transactions, and risk management (Abubakar and Gani 2013; He et al. 2021). Thus, the financial system’s role in economic progress has attracted considerable attention since the debut of the endogenous growth theory, which considers savings as an endogenous variable in the growth model.

The theoretical foundation of the role of financial system development in economic growth can be traced to the seminal study by Schumpeter (2017), which posits that a well-functioning financial system spurs technological innovation and promotes economic growth by enabling efficient allocation of financial resources (savings, credits, and loans) to pragmatic entrepreneurs and funding productive investment. The study further shows that the development of the financial sector enables efficient and effective management and channeling of savings and credits, as well as facilitates productive investment leading to economic growth (Arthur 2017).

Following Schumpeter (1911), Becker and Knudsen (2002) explains that in the finance-led growth hypothesis, the relationship between finance and economic growth continues to attract theoretical and empirical discourse. In this regard, the theoretical literature identifies five major channels through which the financial system drives economic growth. First, the financial system channels investment funds to the most productive uses, which bring the highest return on investment and thus promote economic growth and development (Zhang et al. 2019; Cheng and Hou 2020; Byrska et al. 2021; Babatunde and Oyedokun 2022). The second channel considered in the theoretical literature is the risk-management role, which suggests that financial institutions and markets enable firms (investors) to diversify their portfolios and increase liquidity, thereby reducing risk and stimulating economic growth (Chen et al. 2021; Lahouel et al. 2022). The third channel is financial mediation. The financial system facilitates transactions by ensuring efficient financial mediation, which enables the effective transfer of funds from surplus to deficit units, leading to an increase in economic activities (Rousseau and Wachtel 2011; Appiah-Otoo et al. 2022). The fourth channel through which the financial system stimulates economic growth involves fostering entrepreneurship development, innovation, and the adoption of new technologies (Pradhan et al. 2019). Finally, a sound financial system provides incentives for corporate control, which promotes economic growth (Levine 2018, 2021). In addition, theoretical studies, including those by Atje and Jovanovic (1993) and Cooray (2010), adopted the neoclassical growth theory by augmenting the Mankiw et al. (1992) growth model with financial (stock) market indices to explain the role of the financial system in economic growth. Following early theoretical propositions, several studies explain the channels through which the financial system influences economic growth. For instance, Levine (2004) proposed that financial development facilitates economic growth via five channels: the production of information and capital allocation, improvement of risk management, enforcement of corporate governance by monitoring firms, polling savings, and facilitating exchange of goods and services.

The financial system plays a vital role in economic growth and development; nonetheless, the empirical literature on the finance-led growth hypothesis is divided into five hypotheses (Saqib 2015). The supply leading hypothesis (Patrick 1996) posits that financial development stimulates economic growth by increasing the delivery of financial services and propelling economic activities. Earlier proponents of this hypothesis (Goldsmith 1958; McKinnon 1973; Shah 1973; King and Levine 1993; Patrick 1996) posited that financial development has a positive impact on economic growth. These pioneering studies used the value of the financial intermediary as it relates to the GDP as an indicator of development in financial status. For instance, Levine et al. (1999) studied the empirical relationship between stock market development and submitted a strong positive bond between financial development and economic growth and found that financial factors were a core part of the growth process. Levine (2004) also evaluated the relationship between financial development and economic growth and found a robust correlation between different financial indicators and economic growth. Their studies also showed that it was possible to correctly predict the subsequent rate of economic growth using the initial level of financial depth after controlling for other growth-enhancing factors.

Recent empirical studies (Chaudhry et al. 2019; Asanović 2020; Khan et al. 2021; Alhassan et al. 2022; Nguyen et al. 2022) confirm the positive association between financial development and economic growth in different individuals and groups of economies. For example, the maximum entropy bootstrap approach was use by Khan et al. (2019) to test the relationship between development in financial status and growth in Pakistan’s economy. The findings of this study indicate that the depth of financial status and rate of interest positively influence economic growth in the long term. Although the correlation between growth and financial development is positive, it is found to be insignificant in the short run. They concluded that financial development determines economic growth. Puatwoe and Piabuo (2017) also found a positive relationship between financial development and economic growth in both the short and long term for Cameroon. Moreover, recent studies have found that financial development efficiently strengthens the mobilization and utilization of resources and thus, propels economic growth (Ibrahim and Alagidede 2018; Malarvizhi et al. 2019; Mohieldin et al. 2019).

A panel data study (Chaudhry et al. 2019) revealed that financial development, human capital, and technology have positive impacts on economic development in South Asia. Similarly, Alhassan et al*.* (2022) use a comprehensive measure of financial development (financial development index) and find that financial development spurs economic growth in Asia. The study further revealed that the development of financial institutions has a greater impact than the development of financial markets on the economic growth of upper-middle-income countries, while the reverse is the case for lower-middle-income countries in the Asian continent. Olayungbo and Quadri (2019) also found unidirectional causality from financial development to economic growth in sub-Saharan Africa.

The second strand of literature on the finance-growth nexus affirmed that financial development has a negative impact on economic growth. According to studies in this category (see Berkes et al. 2012; Philippon and Reshef 2013; Iwanicz-Drozdowska et al. 2019), due to globalization, financial development makes the domestic economy vulnerable to external financial crises and shocks, which have a negative effect on economic growth. Specifically, in an international cross-country study, Cheng et al*.* (2021) reveal that the relationship between financial development and economic growth is consistently negative.

Another strand of the literature is the demand-following hypothesis, which implies that economic growth stimulates demand for more financial services, and thus leads to the development of financial institutions and markets (see Magaji et al. 2021). However, other studies belong to the feedback causality hypothesis, which posits a mutual causal relationship between financial development and economic growth. Finally, the neutrality of the financial system in terms of economic growth has also received some submissions. Some studies (e.g., Williams 2018) revealed that financial system development does not have a significant impact on economic growth. The current study aligns with the supply leading hypothesis, and the possibility of the negative impact of financial variables on economic growth is also recognized.

Based on the extant studies discussed above, the hypotheses developed in this study regarding finance-led growth are as follows.

#### Hypothesis 3

There is a positive relationship between savings and economic growth.

#### Hypothesis 4

There is a positive relationship between credits and economic growth.

### Export-led growth hypothesis

Exports play an important role in economic development. Exports are also considered a key stimulus for augmenting domestic production through the efficient utilization of resources (Guntukula 2018). Moreover, the theories by Smith (1776) and Ricardo (1817) posit that countries can benefit from trade by producing and exporting goods for which their resources are best suited. Furthermore, the export-led growth hypothesis has gained prominence.

Export-led growth hypothesis posits that exports play a crucial role in economic growth. Exports ensure the creation of positive externalities-job creation in the domestic market (Abosedra and Tang 2019), increased international cooperation and transfer of technology (Cai et al. 2020), improved productivity and innovation, reduced poverty (World Bank 2022a), and export-oriented industrialization. Many studies support the export-led hypothesis in several economies worldwide (Ahmad et al. 2018; Ali and Li 2018; Mao et al. 2019; Felipe and Lanzafame 2020). However, Edo et al. (2020) reported a negative impact of external debt and exports on economic growth in sub-Saharan Africa. Several studies have also documented bidirectional causality between economic growth and exports (Kalaitzi and Cleeve 2018; Reddy 2020). Furthermore, studies assert that exports promote employment, the development of enterprises, and advancement in technological know-how (Babalola et al. 2012; Kumari and Malhotra 2014). We investigate the impact of exports on the economic growth of selected Arab economies based on the following hypothesis:

#### Hypothesis 5

There is a positive relationship between export ratio and economic growth.

The extant literature on the determinants of economic growth has acknowledged capital, labor, financial variables (savings, credits, and investment), and exports as drivers of economic growth in any economy. These theoretical studies provide a theoretical underpinning for the current study. Against this backdrop, this study integrates the theoretical postulations of traditional (neoclassical and endogenous) growth theories, the finance- and export-led hypotheses, to evaluate the relationship between economic growth, labor force participation, exports, savings, and credit in Arab economies. With more emphasis on financial variables, this is to identify the channel(s) that are viable for stimulating economic growth in the region as a whole and the individual economies in the region.

## Data, model, and methodology

### Data

The time period of this study was selected based on the latest available data for Arab economies from 2001 to 2020. The dependent and independent variables in the study are selected based on previous studies discussed in the literature review section. In this study, we used the 2001–2020 period annual data for the selected Arab economies: Algeria, Bahrain, Egypt, Iraq, Kuwait, Lebanon, Libya, Morocco, Tunisia, Saudi Arabia, and Jordan. We use the annual growth rate of real GDP per capita as a proxy for economic growth. Credit for the private sector and the ratio of savings are proxies for financial development. The exports of goods and services represent the value of all goods and other market services provided to the rest of the world. Finally, the labor force participation rate is the proportion of the working-age population who are either working or actively looking for work. The data used in this study were obtained from the World Bank Development Data, Bank of Africa, and International Monetary Fund (IMF). All data were used in logarithmic form.

### Model

This study used a simple but explanatory single-equation model to determine economic growth. The model also uses a socioeconomic indicator to provide clear policy direction. The model used in this study is as follows:

The annual growth rate of real GDP per capita ***(lngdp)* = **credit for private sectors ***(lncrd)*** + ratio of savings ***(lnsav)*** + ratio of export ***(lnexp)*** + real GDP per labor force participant ***(lnlab).***

The regression model for this study is as follows^1^:

1 $$\ln gdp = \beta_{0i} + \beta_{1i} \ln \exp_{it} + \beta_{2i} \ln sav_{it} + \beta_{3i} \ln lab_{it} + \beta_{4i} \ln crd_{it} + \varepsilon_{it}$$

where $\beta_{0i}$, $\beta_{1i}$, $\beta_{2i}$, $\beta_{3i}$, and $\beta_{4i}$ are the coefficients of constant, lnexp, lnsav, lnlab, and lncrd, respectively. $\varepsilon_{it}$ is the error term.

**Table 1:** Cross-section dependence and slope homogeneity tests results

Variables	CD_LM1_	CD_LM2_	CD
lngdp	611.136*	53.025*	23.605*
lncrd	526.364*	44.942*	13.504*
lnsav	273.135*	20.798*	10.179*
lnexp	299.637*	23.325*	11.596*
lnlab	388.532*	31.801*	7.773*
Model	205.698*	14.368*	0.164

Slope homogeneity	Test statistics	*P*-value	
$\hat{\Delta }$	10.483*	0.000	
$\hat{\Delta }_{adj}$	12.403*	0.000	

*Indicates the rejection of the null hypothesis at a 1% significance level

The annual growth rate of real GDP per capita ***(lngdp)* = **credit for private sectors ***(lncrd)*** + ratio of savings ***(lnsav)*** + Ratio of export ***(lnexp)*** + real GDP per labor force participant ***(lnlab).***

### Methodology

This study used three different tests to investigate the relationship between cross-sectional units. The first test is the LM test (Breusch and Pagan 1980), and the test statistics used for this test are calculated as follows:

2 $$CD_{LM1} = \,T\sum\limits_{i = 1}^{N - 1} {\sum\limits_{j = i + 1}^{N} {\hat{\rho }_{ij}^{2} } }$$

Pesaran (2004) developed another cross-sectional dependency test that could be used for larger values of N and T. This test statistic, which is called CDLM2, is calculated as follows:

3 $$CD_{LM2} = \,\sqrt {\frac{1}{N(N - 1)}} \sum\limits_{i = 1}^{N - 1} {\sum\limits_{j = i + 1}^{N} {(T\hat{\rho }_{ij}^{2} } } - 1)$$

Finally, Pesaran (2004) stated that the CDLM2 test did not provide reliable results in cases where N was greater than T and developed a CD test that could be used in this case.

4 $$CD = \,\sqrt {\frac{2T}{{N(N - 1)}}} \left( {\sum\limits_{i = 1}^{N - 1} {\sum\limits_{j = i + 1}^{N} {\hat{\rho }_{ij} } } } \right)$$

where T, N, and $\hat{\rho }_{ij}$ indicate the time, cross-section dimensions, and squares of the estimated pairwise correlation of the residuals, respectively, for the three test statistics. The null hypotheses of the cross-sectional dependency tests state no cross-sectional dependence, whereas the alternative hypotheses indicate cross-sectional dependence. We investigated the slope homogeneity for Model 1 using Pesaran and Yamagata (2008) delta tests based on the modified Swamy (1970) statistic ($\tilde{S}$).

5 $$\hat{\Delta } = \sqrt N \left( {\frac{{N^{ - 1} \tilde{S} - k}}{{\sqrt {2k} }}} \right)\;{\text{and}}\;\hat{\Delta }_{adj} = \sqrt N \left( {\frac{{N^{ - 1} \tilde{S} - E(\tilde{z}_{\^i t} )}}{{\sqrt {{\text{var}} (\tilde{z}_{\^i t} )} }}} \right)$$

where $E(\tilde{z}_{\^i t} ) = k,\,\,{\text{var}} (\tilde{z}_{\^i t} ) = 2k(T - k - 1)/T + 1$. Both test statistics use the homogeneity null hypothesis versus the alternative heterogeneity hypothesis. Nazlioglu and Karul (2017) developed an LM-type panel stationarity test that considers smooth structural breaks. The following models are used for this test, also called the panel version of the Fourier Kwiatkowski–Phillips–Schmidt–Shin (KPSS) test:

6 $$\begin{aligned} & y_{i,t} = \alpha_{i} (t) + r_{i,t} + \lambda_{i} F_{t} + \varepsilon_{i,t} \\ & r_{i,t} = r_{i,t - 1} + u_{i,t} \\ & \alpha_{i} (t) = a_{i} + b_{i} t + \gamma_{1i} \sin \left( {2\pi kt/T} \right) + \gamma_{2i} \cos \left( {2\pi kt/T} \right) \\ \end{aligned}$$

where $\alpha_{i} (t)$,$F_{t}$, $r_{i,t}$, and $\lambda_{i}$ are the deterministic term, unobserved common factor, random walk process, and loading weight, respectively. The Fourier panel test statistic is calculated by averaging the Fourier KPSS test statistics developed by Becker et al. (2006).

7 $$FP(k) = \frac{1}{N}\sum\limits_{i = 1}^{N} {\eta_{i} (k)}$$

The test statistic for the panel stationarity (Fourier PKPSS) test is defined as follows:

8 $$FZ(k) = \frac{{\sqrt N \left( {FP(k) - \xi (k)} \right)}}{\zeta (k)}N(0,1)$$

where $\xi (k)$ and $\zeta (k)$ are the mean and variance of individual statistics, respectively. For the Fourier PKPSS test, the null hypothesis shows stationarity, whereas the alternative hypothesis shows the unit root. We also use the cross-sectionally augmented Im-Pesaran-Shin (CIPS) panel unit root test proposed by Pesaran (2007). The CIPS test considers cross-section dependence and individual dynamic specifications in each regression. Pesaran (2007) used the following statistic to test the unit root:

9 $$CIPS(N,T) = N^{ - 1} \sum\limits_{i = 1}^{N} {t_{i} } (N,T)$$

where $t_{i} (N,T)$ is the cross-sectionally augmented Dickey-Fuller (CADF) statistic for each cross-section unit. The null hypothesis shows nonstationarity, whereas the alternative hypothesis shows stationarity. Westerlund and Edgerton (2008) developed a panel cointegration test that considers cross-sectional dependence and heterogeneous slopes. The most important feature of this test that distinguishes it from other panel cointegration tests is that it allows for structural breaks. Thus, reliable results were obtained in the presence of structural breaks. Westerlund and Edgerton (2008) suggested using the following model for the panel cointegration test:

10 $$y_{it} = A_{i} + \mu_{i} t + \alpha_{i} D_{it} + x_{it}^{{\prime }} B_{i} + \left( {D_{it} x_{it} } \right)^{{\prime }} b_{i} + \varepsilon_{1it} ,\;x_{it} = x_{it - 1} + \varepsilon_{2it}$$

where $x_{it}$ is a set of independent variables, $D_{it}$ is a dummy variable for structural breaks, and $\varepsilon_{it}$ is an error term. The equations that allow cross-section dependence between unforeseen conjoint factors for this test are as follows:

11 $$\begin{aligned} & \varepsilon_{2it} = \rho_{i}^{{\prime }} F_{t} + m_{it} , \\ & F_{it} = \omega_{j} F_{jt - 1} + n_{it} , \\ & \phi_{i} (L)\Delta m_{it} = \phi_{i} \Delta m_{it - 1} + \rho_{it} ,\;\phi_{i} (L) = 1 - \sum {\phi_{i,j} } L_{j} \\ \end{aligned}$$

where $\rho_{i}$ is a factor loading parameter vector, and $F_{t}$ is an unforeseen conjoint factor. To overcome the cross-section dependency problem, Westerlund and Edgerton (2008) obtained residuals as follows:

12 $$\hat{S}_{it} = y_{it} - \hat{A}_{i} - \hat{\mu }_{i} t - \hat{\alpha }_{i} D_{it} - x_{it}^{{\prime }} \hat{B}_{i} - \left( {D_{it} x_{it} } \right)^{{\prime }} \hat{b}_{i} - \hat{\lambda }_{i}^{{\prime }} \hat{F}_{t}$$

They proposed the following model for a robust test statistic against serial correlation.

13 $$\Delta \hat{S}_{it} = {\text{constant + }}\phi_{i} \hat{S}_{it - 1} + \sum\limits_{j = 1}^{t} {\phi_{ij} } \Delta \hat{S}_{it - j} + {\text{error}}$$

Westerlund and Edgerton (2008) test statistics are as follows:

14 $$Z_{\tau } = \frac{{\hat{\phi }_{i} }}{{SE\left( {\hat{\phi }_{i} } \right)}},\;Z_{\theta } = T\hat{\phi }_{i} \left( {\frac{{\hat{\omega }_{i} }}{{\hat{\sigma }_{i} }}} \right)$$

where $\hat{\phi }_{i}$ and $\hat{\sigma }_{i}$ are the least-squares estimates of $\phi_{i}$ in Eq. (13), and the estimated standard error from Eq. (12), respectively; and $\hat{\omega }_{i}$ is defined as follows:

15 $$\hat{\omega }_{i}^{2} = \frac{1}{T - 1}\sum\limits_{{j = - M_{i} }}^{{M_{i} }} {\left( {1 - \frac{j}{{M_{i} + 1}}} \right)} \sum\limits_{t = j + 1}^{T} {\Delta \hat{S}_{it} } \Delta \hat{S}_{it - j}$$

where $M_{i}$ is the kernel-bandwidth parameter. We use two estimators to estimate long-term parameters. The mean group estimators follow the same two-step methodology. In the first step, a group-specific regression was estimated. In the second step, the estimated coefficients across the groups were averaged. The mean group estimators used the following models:

16 $$\begin{aligned} & y_{it} = \beta_{i} x_{it} + u_{it} \\ & x_{it} = \alpha_{2i} + \lambda_{i} f_{t} + \gamma_{i} g_{t} + \varepsilon_{it} \\ & u_{it} = \alpha_{1i} + \lambda_{i} f_{t} + e_{it} \\ \end{aligned}$$

where $\beta_{i}$, $u_{it}$, and $e_{it}$ are the country-specific slopes, unobservables, and error terms, respectively. Pesaran (2006) proposed the common correlated effects mean group estimator (CCEMG) to estimate long-term parameters. The CCEMG estimator considers cross-sectional dependence, time-variant unobservables, and identification problems. Eberhardt and Teal (2010) proposed the augmented mean group estimator (AMG), based on production function estimation, as an alternative to the CCEMG estimator. The AMG estimator follows a three-step procedure: In the first step, a pooled regression model with year dummies was estimated using the first difference least squares method, and the coefficients for the year dummies were obtained. These represent an estimated between-group mean of the evolution of the unobservable total factor productivity (TFP) over time. In the second step, we estimate the group-specific regression model using the TFP estimates obtained in the first step. Finally, group-specific model parameters were averaged across the panel. In this study, we used second-generation tests that considered the cross-sectional dependence of panel data models. In addition, unlike other methodologies in the literature, we did not ignore structural breaks. Accordingly, we robust our results using the unit root and cointegration tests, allowing for structural breaks.

## Empirical results

The proper analytical approach begins by establishing whether the panel has a cross-sectional dependency. The null hypothesis posits that the series is cross-sectionally independent, whereas the alternative hypothesis suggests that the series is cross-sectionally dependent. The findings of the cross-sectional reliance check are used to reject the null hypothesis and support the alternative assertion that there is cross-sectional dependency within the data series.

Table 1 presents the results of the cross-sectional dependency. The results indicate a cross-sectional dependency in the series. This means that the null hypothesis of cross-sectional independence in the series is rejected. The slope homogeneity test is presented in the second part of Table 1. The results indicated a slope homogeneity in the model. Hence, based on the above analysis, the panel heterogeneity model was used to analyze the relationship between dependent and independent variables.

Table 2 presents the unit root and stationarity tests used for the cross-sectional series. The results of the Fourier PKPSS test indicated that all variables rejected the null hypothesis of stationarity in the series. This means that all variables are stationary at the first level. The CIPS panel unit root test results show that all variables are stationary at first differences, whereas they have a unit root at level. Accordingly, all variables were I (0).

**Table 2:** Unit root and stationarity tests results

	FPKPSS stationarity test			CIPS unit root test
Variables	Level	First difference	Level	First difference
lngdp	3.459*	1.143	− 2.052	− 3.509*
lncrd	5.811*	1.625	− 2.110	− 4.571*
lnsav	3.832*	-0.679	− 1.909	− 4.367*
lnexp	14.57*	0.496	− 1.701	− 4.480*
lnlab	21.66*	1.212	− 1.888	− 2.921*

*Indicates the rejection of the null hypothesis at a 1% significance level. The null hypothesis of the FPKPSS test is stationarity

Table 3 presents the Westerlund and Edgerton (2008) panel cointegration test. To show the long-term relationship between the stated variables in the study, we use the Westerlund and Edgerton’s (2008) panel cointegration under the null hypothesis of no cointegration by McCoskey and Kao (1998). The Lagrange multiplier test considers potential correlation, both inside and across cross-sections, and can significantly minimize the distortions of the applied asymptotic test. This result indicates that economic growth is cointegrated with explanatory variables or that there is a long relationship among them. As per the above test, such as cross-sectional dependency, unit root, and Westerlund and Edgerton’s (2008) panel cointegration results, we proceed to examine the long-term relationship between the variables (Table 4).

**Table 3:** Panel cointegration test results

Test	Test Stat	*P*-value
Westerlund and Edgerton (2008)		
Z_τ_	− 4.867*	0.000
Z_Ø_	− 1.396***	0.081

* and ***Indicate the rejection of the null hypothesis at 1% and 10% significance levels, respectively

**Table 4:** Long-run estimation results

Countries		Lnexp	lnsav	lnlab	Lncrd
Algeria	AMG	0.218	0.219	0.843*	− 0.131
	CCEMG	0.427	− 0.154	0.581***	− 0.149
Bahrain	AMG	− 0.150***	− 0.098	0.283**	− 0.526*
	CCEMG	0.036	0.007	0.492***	− 0.290
Egypt	AMG	− 0.731*	0.163*	0.154	− 0.170
	CCEMG	− 0.232	0.153*	− 1.007**	− 0.132
Iraq	AMG	0.020	− 0.064**	0.054	0.080
	CCEMG	− 0.490***	− 0.062	− 0.074	− 0.200
Kuwait	AMG	− 2.279*	1.307*	0.419**	− 0.828*
	CCEMG	− 1.326*	0.552**	0.170	− 0.589*
Lebanon	AMG	− 0.385**	− 0.023	− 0.058	− 0.413
	CCEMG	− 0.389**	0.022	− 1.016	− 0.816
Libya	AMG	− 0.679*	− 0.196	− 0.927	− 0.899*
	CCEMG	− 0.537	− 0.131	− 0.568	− 0.877*
Morocco	AMG	− 0.354	0.565	− 0.011	− 0.279
	CCEMG	0.184	0.115	− 0.023	− 0.055
Tunisia	AMG	− 0.268*	0.076	− 0.710*	− 0.168
	CCEMG	− 0.227***	0.162***	− 0.391	− 0.028
Saudi Arabia	AMG	− 0.291	0.513***	− 0.787	0.083
	CCEMG	0.040	0.595	− 0.714	0.126
Jordan	AMG	− 2.162*	0.196	− 0.954***	− 0.383*
	CCEMG	− 0.419***	0.098	0.799**	0.053
Panel	AMG	− 0.334**	0.158	− 0.151	− 0.291**
	CCEMG	− 0.224***	0.038	− 0.160	− 0.178

*, **, and ***Indicate the rejection of the null hypothesis at 1%, 5%, and 10% significance levels, respectively

We used two estimators (AMG and CCEMG) to obtain robust results. We used both individual and panel country analyses to obtain precise findings. We find that the share of exports has a significantly negative effect on economic growth in countries such as Kuwait, Lebanon, Tunisia, and Jordan. Similarly, panel analysis also asserts a negative relationship between exports and economic growth in the selected Arab countries. Interpretatively, a 1% increase in exports leads to a -0.334 and -0.224 decrease in economic growth, as per the results of AMG and CCEMG estimators. This is possible in the absence of export promotion policies, which not only hinder international trade but also impede the economic growth of selected Arab economies. This finding is in line with the conclusion drawn by Bakari (2017) who found that exports and domestic investments have a negative impact on economic growth in the long run. Therefore, we reject our *first hypothesis* that economic growth is positively influenced by exports in selected Arab economies. Generally, trade surplus helps a country’s economic growth. When a country’s exports rise, it shows a high level of output from its factories and industrial facilities as well as a growth in the number of employees employed to keep these businesses going. When a corporation exports a large number of commodities, it also brings money to the country, which promotes consumer spending and contributes to economic growth. When a company exports a considerable number of goods, it brings money to the country, which encourages consumer spending and contributes to economic growth (Davis and Tilton 2005). A strong economy is one in which exports and imports increase. This usually signifies economic strength and a long-term trade surplus or deficit. If exports decline substantially but imports rise, this may signal that the home economy is doing better than international markets (Ihrig et al. 2010). These findings are similar to those of previous studies (Jaffee 1985). However, there is a positive relationship between the ratio of savings and economic growth in few countries, such as Egypt, Kuwait, and Saudi Arabia. This means that a 1% increase in savings leads to a 0.16% increase in economic growth in Egypt. Similarly, panel analysis asserts a positive relationship between savings and economic growth in selected Arab countries. Interpretatively, a 1% increase in exports leads to a 0.158 and 0.038 increase in economic growth as per the results of AMG and CCEMG estimator farming. Everything being equal, a higher saving rate leads to an increase in physical capital over time, allowing the economy to produce more goods and services (Beck et al. 2000).

Likewise, labor force participation is positively associated with economic growth in some individual countries, such as Algeria and Bahrain. However, we observe that the labor force participation rate has a negative impact on the economic growth of other Arab countries. Similarly, the panel analysis shows a negative relationship between labor force participation and economic growth. Interpretatively, a 1% increase in labor force participation leads to a − 0.151 and − 0.160 increase in economic growth, as per the results of AMG and CCEMG estimators, respectively. This could be possible because, in this era of global economic growth, it is possible to make substantial investments in the advancement of technological know-how instead of the labor force. This indicates that a skilled labor force enhances economic growth. This is in line with the conclusions of Shahbaz et al. (2018). Furthermore, financial development is a key factor that positively affects economic growth. However, we find a significant negative relationship between financial development and economic growth in countries such as Kuwait and Libya. Moreover, the panel analysis posits a negative relationship between financial development and economic growth in Arab economies. The unfavorable impact of financial development could be due to the inefficient operation of the financial sector in the country. Moreover, studies have documented that beyond a certain threshold level, lending to the private sector deteriorates economic growth (Law and Singh 2014). Thus, we reject our *second hypothesis* that financial development positively augments the economic growth of selected Arab economies. This study is similar to the findings of Al-Yousif (2002).

## Concluding remarks and recommendations

Achieving the United Nations Sustainable Development Goals (UNSDGs-8) is seemingly a huge task for many economies across the globe, and Arab economies are not excluded from this list of economies that are facing challenges. The traditional growth model outlines capital and labor stock accumulation as catalysts for economic growth. However, over the year, most economies have explored other routes or alternatives and complementary growth drivers, such as the finance- and tourism-led growth hypotheses, export-led growth argument, and energy-led growth hypothesis. The need for sustainable growth in the era of many economic tumors is a necessary pursuit for most economies. The present study focuses on Arab economies using recent and robust panel econometrics tools such as the Westerlund and Edgerton cointegration test, AMG complemented with the CCEMG model, and Fourier KPSS test for the robustness of analysis and policy inferences.

Our results highlight the cointegration relationship between the outlined study variables over the sampled period. The key findings of this study are as follows:

(i) The share of exports is negatively and significantly associated with economic growth in countries such as Kuwait, Lebanon, Tunisia, and Jordan. Similarly, panel studies affirm the negative relationship between exports and economic growth in these countries. This finding affirms the propositions of the export-led growth hypothesis, which acknowledges the importance of exports as drivers of economic growth. However, the negative association between export share and economic growth implies that the nature of exports in Arab economies does not spur economic activities. Natural resources and primary commodities are the main exports of these countries. For instance, precious stones and metals are the main export products in Lebanon, whereas Kuwait and Tunisia mainly export mineral fuel (oil) and petroleum products, respectively, which (UNCTAD 2021). could hinder economic growth owing to the neglect of other sectors and the substantial imports to meet domestic demand. This highlights that the aforementioned countries need to implement export promotion strategies that engender their export of finished products to improve their export-to-import share. It is well established that a trade surplus translates into increased trade volume and, by extension, creates a direct and indirect effect of macroeconomic impact and economic growth in the long run. Thus, from a policy perspective, there is a need for economies such as Kuwait, Lebanon, Tunisia, and Jordan to invest massively in export-oriented industrialization by supporting companies and providing credit facilities for the export sector of their economies. This will help eliminate trade deficits and create trade surpluses in these countries.
(ii) Furthermore, we observed a positive relationship between the ratio of savings and economic growth in few countries such as Egypt, Kuwait, and Saudi Arabia. This finding is consistent with the supply leading strand of the finance-led growth hypothesis. This finding implies that an increased share of savings increases economic growth. A higher saving rate in these countries results in more investment and accumulation of capital over time, leading to the production of more goods and services and economic growth. Therefore, these countries should encourage savings by reducing taxes, creating jobs, and supporting entrepreneurship. All these will reduce poverty and improve economic savings. However, the significantly negative relationship between savings and economic growth in Iraq provides interesting insights. This reveals that savings negatively affects economic growth in the country. A plausible explanation for this can be that savings in Iraq are not converted into investment because of the age-long effect of terrorism in the country. The country does not have an investment-friendly environment. Hence, savings are eventually either invested abroad, used for the consumption of foreign goods, or left idle because of a weak financial system. In any of these cases, savings have a negative impact on Iraq’s economic growth.
(iii) Mixed results originate from the relationship between labor per capita and economic growth among the investigated countries. As per the panel, there is no significant relationship between labor force participation and economic growth. This outcome is contrary to the conventional classical growth model, which posits that labor is a primary determinant of growth. However, the findings established a positive and significant relationship between the labor force and economic growth in Algeria, Bahrain, Egypt, Iraq, and Kuwait, which demonstrate a positive and significant relationship with economic growth. The policy taken from this result is that, for these countries to stimulate economic growth, they should embark on policies that will improve labor force participation by both genders in the labor force and the quality of labor through education and training activities.

In summary, there is a need for the Arab bloc to open its economies to the rest of the world to gain from exports. There is a need to participate in the private sector’s involvement in the economic growth trajectory, as seen in our baseline regression. Labor and capital remain integral to economic growth, but much more effort is required in the art of the investigated economies to attain the anticipated SDG-8.

This study examines the relationship between GDP per capita, credit for the private sector, and the ratio of export real GDP per labor force participants in selected Arab economies. Our study does not claim to be exhaustive for all the pertinent drivers of sustainable growth. Further studies can provide an appreciable contribution by investigating the role of institutions in the sustainable growth of Arab nations. In addition, considering this issue at the firm level can provide further insights and policy directions. Finally, our study leaves room for future research to explore other Arab economies for growth catalysts, such as energy and foreign direct investment (FDI)-led growth.

## Figures and Tables

**Fig. 1:**
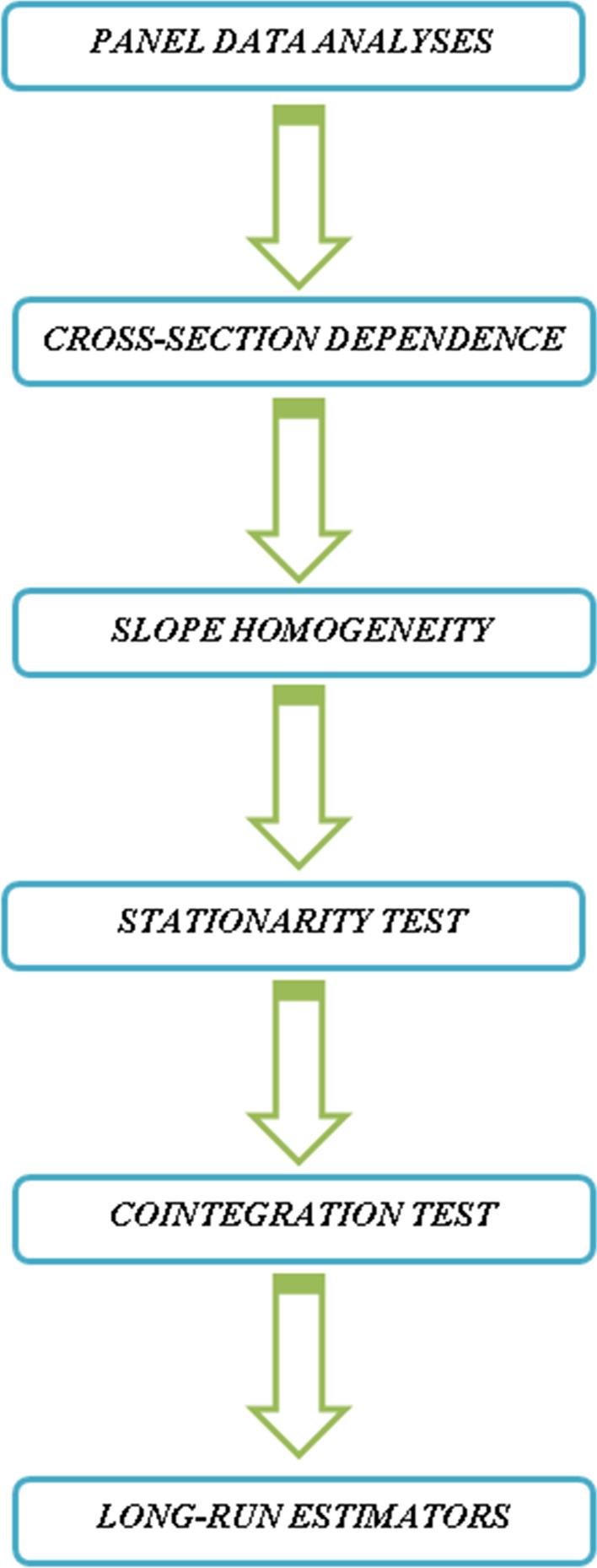
The steps of the methodology

## Data Availability

The datasets used and/or analyzed during the current study are available from the corresponding author on reasonable request.

## Appendix

See Fig. 1, Table 5.

**Table 5:** Correlation matrix

	EXP	SAV	LAB	CRD
EXP	1.000	0.493	0.124	− 0.148
SAV	0.493	1.000	0.176	− 0.432
LAB	0.124	0.176	1.000	− 0.131
CRD	− 0.148	− 0.432	− 0.131	1.000

## Abbreviations

GDP: Gross Domestic Product
UNSDG: United Nations Sustainable Development Goals
KPSS: Kwiatkowski–Phillips–Schmidt–Shin
AMG: Augmented Mean Group
CCEMG: Common Correlated Error Mean Group
FDI: Foreign Direct Investment

## Footnotes

1 We checked the multicollinearity of the model, and the results are reported in Table A1 in the Appendix.

## References

[CR1] Abosedra S, Tang CF (2019). Are exports a reliable source of economic growth in MENA countries? New evidence from the rolling Granger causality method. Empir Econ.

[CR2] Abraham KG, Kearney MS (2020). Explaining the decline in the US employment-to-population ratio: a review of the evidence. J Econ Lit.

[CR3] Abubakar A, Gani IM (2013). Impact of banking sector development on economic growth: another look at the evidence from Nigeria Editor BORJ impact of banking sector development on economic growth: another look at the evidence from Nigeria. J Bus Manag Soc Sci Res.

[CR4] Ahmad F, Draz MU, Yang S-C (2018). Causality nexus of exports, FDI and economic growth of the ASEAN5 economies: evidence from panel data analysis. Science.

[CR5] Alhassan H, Kwakwa PA, Donkoh SA (2022). The interrelationships among financial development, economic growth and environmental sustainability: evidence from Ghana. Environ Sci Pollut Res.

[CR6] Ali G, Li Z (2018). The international trade journal exports-led growth or growth-led exports in the case of China and Pakistan: an empirical investigation from the ARDL and Granger causality approach. Int Trade J.

[CR7] Alkin E (2016). The problematic of savings: the case of Turkey as an emerging market economy. Aurum J Soc Sci.

[CR8] Al-Yousif YK (2002). Financial development and economic growth: another look at the evidence from developing countries. Rev Financ Econ.

[CR9] Appiah-Otoo I (2022). Foreign aid-economic growth Nexus in Africa: does financial development matter?. Int Econ J.

[CR207] Arthur MS (2017) Dual enrollment as a path to higher education in Oregon (Doctoral dissertation, Concordia University (Oregon))

[CR10] Asanović Ž (2020). Essay on finance-growth Nexus. J Central Bank Theory Pract.

[CR11] Atje R, Jovanovic B (1993). Stock markets and development. Eur Econ Rev.

[CR12] Azar S, Bolbol A, Mouradian A (2018). Private savings in the Arab countries: empirical analysis and policy implications. Int J Econ Financ.

[CR13] Azar SA, Bolbol A, Mouradian A (2018). Private savings in the Arab countries: empirical analysis and policy implications. Int J Econ Finance.

[CR14] Babalola SJ, Dogon-Daji SDH, Saka JO (2012). Exports, foreign direct investment and economic growth: an empirical application for Nigeria. Int J Econ Finance.

[CR15] Babatunde O, Oyedokun GE (2022). Financial intermediation and nigerian economic growth. Int J Soc Sci Manag Rev.

[CR16] Baerlocher D, Parente SL, Rios-Neto E (2021). Female Labor Force Participation and economic growth: accounting for the gender bonus. Econ Lett.

[CR17] Bakari S (2017). The relationship between export, import, domestic investment and economic growth in Egypt: empirical analysis. EuroEconomica.

[CR18] Beck T, Levine R, Loayza N (2000). Finance and the sources of growth. J Financ Econ.

[CR19] Becker MC, Knudsen T (2002). Schumpeter 1911: farsighted visions on economic development. Am J Econ Sociol.

[CR20] Becker R, Enders W, Lee J (2006). A stationarity test in the presence of an unknown number of smooth breaks. J Time Ser Anal.

[CR21] Berkes EG, Panizza U, Arcand J-L (2012). Too much finance?. IMF Work Pap.

[CR22] Breusch TS, Pagan AR (1980). The Lagrange multiplier test and its applications to model specification in econometrics. Rev Econ Stud.

[CR23] BTI (2022) BTI 2022: Middle East and North Africa: BTI 2022. https://bti-project.org/en/reports/regional-dashboard/ENA?&cb=00000. Accessed 4 Aug 2022

[CR24] Byrska D, Krawiec A, Szydłowski M (2021). Dynamics of a simple endogenous growth model with financial intermediation. Macroecon Dyn.

[CR25] Cai Y, Wu G, Zhang D (2020). Does export trade promote firm innovation?. Ann Econ Financ.

[CR26] Chaudhry IS, Sabir S, Gulzar F (2019). Technology and economic growth: role of financial development in South Asian countries. Rev Econ Dev Stud.

[CR27] Chen Y, Kusuma KE, Sivakumar V (2021). Investigation of finance industry on risk awareness model and digital economic growth. Ann Oper Res.

[CR28] Cheng S-Y, Hou H (2020). Do non-intermediation services tell us more in the finance-growth nexus?: causality evidence from eight OECD countries. Appl Econ.

[CR29] Cooray A (2010). Do stock markets lead to economic growth?. J Policy Model.

[CR30] Daily News Egypt (2022) Arab economies are expected to grow by 5% in 2022: AMF, Daily News. https://dailynewsegypt.com/2022/04/20/778436/. Accessed 4 Aug 2022

[CR31] Davis GA, Tilton JE (2005) The resource curse. In: Natural resources forum, pp 233–242

[CR32] Eberhardt M, Teal F (2010) Aggregation versus Heterogeneity in Cross-Country Growth Empirics*. In: Centre for the Study of African Economies, pp 1–37. https://ora.ox.ac.uk/objects/uuid:adc43db1-daab-434a-9aea-ac149861d6a9/download_file?file_format=pdf&safe_filename=2010-32text.pdf&type_of_work=Working+paper. Accessed 6 Aug 2022

[CR190] Edo S, Osadolor NE, Dading IF (2020) Growing external debt and declining export: The concurrent impediments in economic growth of Sub-Saharan African countries. Int Econ 161:173–187

[CR206] Eftimoski D (2022) Human Capital and economic growth in OECD countries revisited: initial stock versus changes in the stock of human capital effects. Jahrbücher für Nationalökonomie und Statistik 242(1):1–38

[CR33] Felipe J, Lanzafame M (2020). The PRC’s long-run growth through the lens of the export-led growth model. J Comp Econ.

[CR34] Girón A, Kazemikhasragh A (2021). Gender equality and economic growth in Asia and Africa: empirical analysis of developing and least developed countries. J Knowl Econ.

[CR35] Goldsmith RW (1958) The role of financial intermediaries in the financial structure of the American economy. In: Financial intermediaries in the American economy since 1900. Princeton University Press, pp 297–334. http://www.nber.org/books/gold58-1. Accessed 6 Aug 2022

[CR36] Guntukula R (2018). Exports, imports and economic growth in India: evidence from cointegration and causality analysis. Theor Appl Econ.

[CR37] Haque AU (2019). Labor force participation rate and economic growth: observations for Bangladesh. Int J Econ Financ Res.

[CR38] He X (2021). Chinese banking sector: a major stakeholder in bringing fourth industrial revolution in the country. Technol Forecast Soc Change.

[CR39] Hou Y, Karayalcin C (2019). Exports of primary goods and human capital accumulation. Rev Int Econ.

[CR40] Hungwe M, Odhiambo NM (2019). Savings and investment dynamics in South Africa. Acta Universitatis Danubius Economica.

[CR41] Ibrahim M, Alagidede P (2018). Effect of financial development on economic growth in sub-Saharan Africa. J Policy Model.

[CR42] Ihrig J (2010). Some simple tests of the globalization and inflation hypothesis. Int Finance.

[CR43] International Monetary Fund (2016) Economic diversification in oil-exporting Arab Countries, Annual Meeting of Arab Ministers of Finance. Manama, Bahrain. 10.1113/jphysiol.1974.sp010682

[CR44] Islam MR, Haque M (2018). The trends of export and its consequences to the GDP of Bangladesh. J Soc Sci Hum.

[CR45] Iwanicz-Drozdowska M (2019). The role of banks in CESEE countries: exploring non-standard determinants of economic growth. Post-Commun Econ.

[CR46] Jaffee D (1985). Export dependence and economic growth: a reformulation and respecification. Soc Forces.

[CR200] Joshi A, Pradhan S, Bist JP (2019) Savings, investment, and growth in Nepal: an empirical analysis. Financ Innov 5(1):1–13

[CR47] Kalaitzi AS, Cleeve E (2018). Export-led growth in the UAE: multivariate causality between primary exports, manufactured exports and economic growth. Eurasian Bus Rev.

[CR48] Keynes JM (1931). ‘The pure theory of money. A reply to Dr. Hayek. Economica.

[CR49] Keynes JM (1936). The supply of gold. Econ J.

[CR50] Khalid A-I, Hassan H (2022) Weak pass-through of growth to household incomes in Arab countries, Economic Research Forum. https://theforum.erf.org.eg/2022/07/18/weak-pass-growth-household-incomes-arab-countries/. Accessed 4 Aug 2022

[CR51] Khan A, Ahmed M, Bibi S (2019). Financial development and economic growth nexus for Pakistan: a revisit using maximum entropy bootstrap approach. Empir Econ.

[CR52] Khan MK (2021). Moving towards sustainability: how do natural resources, financial development, and economic growth interact with the ecological footprint in Malaysia? A dynamic ARDL approach. Environ Sci Pollut Res.

[CR53] King RG, Levine R (1993). Finance, entrepreneurship, and growth Theory and evidence*. J Monet Econ.

[CR54] Korotayev AV, Issaev LM, Shishkina AR (2015). Female labor force participation rate, Islam, and Arab culture in cross-cultural perspective. Cross Cultural Res.

[CR55] Kou G (2021). Fintech investments in European banks: a hybrid IT2 fuzzy multidimensional decision-making approach. Financ Innov.

[CR56] Kou G, Yüksel S, Dinçer H (2022). Inventive problem-solving map of innovative carbon emission strategies for solar energy-based transportation investment projects. Appl Energy.

[CR57] Kumari D, Malhotra N (2014). Export-led growth in India: cointegration and causality analysis. J Econ Dev Stud.

[CR58] Lahouel B (2022). Financial stability, liquidity risk and income diversification: evidence from European banks using the CAMELS-DEA approach. Ann Oper Res.

[CR301] Law SH, Singh N (2014) Does too much finance harm economic growth? J Banking Financ 41:36–44

[CR59] Levine R (2004) Finance and growth: theory and evidence. National Bureau of Economic Research Working Paper No. 10766

[CR60] Levine R (2018). Finance, growth and economic prosperity. Macroecon Rev.

[CR61] Levine R (2021). Finance, growth, and inequality. IMF Work Pap.

[CR62] Levine R, Loayza N, Beck T (1999). Financial intermediation and growth: causality and causes.

[CR202] Lucas Jr RE (1988) On the mechanics of economic development. J Monetary Econ 22(1):3–42

[CR63] Magaji S, Darma NA, Igwe GU (2021). Testing the supply-leading and demand-following hypothesis for financial development and economic growth-a case of the Nigerian banking system. Global Sci J.

[CR64] Malarvizhi CAN (2019). Financial development and economic growth in ASEAN-5 countries. Article Glob Bus Rev.

[CR65] Mankiw NG, Romer D, Weil DN (1992). A contribution to the empirics of economic growth. Q J Econ.

[CR66] Mao R, Yao Y, Zou J (2019). Productivity growth, fixed exchange rates, and export-led growth. China Econ Rev.

[CR67] McCoskey S, Kao C (1998). A residual-based test of the null of cointegration in panel data. Economet Rev.

[CR68] McKinnon RI (1973). The value-added tax and the liberalization of foreign trade in developing economies: a comment. J Econ Lit.

[CR69] Mohieldin M, Hussein K, Rostom A (2019). On financial development and economic growth in Egypt. J Hum Appl Soc Sci.

[CR70] Nazlioglu S, Karul C (2017). A panel stationarity test with gradual structural shifts: re-investigate the international commodity price shocks. Econ Model.

[CR220] Nguyen HM, Le QTT, Ho CM, Nguyen TC, Vo DH (2022) Does financial development matter for economic growth in the emerging markets? Borsa Istanbul Rev 22(4):688–698

[CR71] Olayungbo DO, Quadri A (2019). Remittances, financial development and economic growth in sub-Saharan African countries: evidence from a PMG-ARDL approach. Financ Innov.

[CR72] Osipian A (2007) Economic growth: education as a factor of production. Available at SSRN 1125313 [Preprint]. https://ssrn.com/abstract=1125313 or 10.2139/ssrn.1125313

[CR73] Patrick HT (1996). Financial development and economic growth in underdeveloped countries. Econ Dev Cultural Change.

[CR74] Pesaran (2007). Journal of Applied Econometrics. J Appl Econ.

[CR75] Pesaran MH (2004) General diagnostic tests for cross section dependence in panels (IZA Discussion Paper No. 1240). Institute for the Study of Labor (IZA) [Preprint]

[CR76] Pesaran MH, Yamagata T (2008). Testing slope homogeneity in large panels. J Econ.

[CR77] Philippon T, Reshef A (2013). An international look at the growth of modern finance. J Econ Perspect.

[CR78] Pradhan R (2019). Attaining economic growth through financial development and foreign direct investment. J Econ Stud.

[CR79] Puatwoe JT, Piabuo SM (2017). Financial sector development and economic growth: evidence from Cameroon. Financ Innov.

[CR80] Reddy KK (2020). Exports, imports and economic growth in India: an empirical analysis. Theor Appl Econ.

[CR81] Ricardo D (1817) On the principles of political economy and taxation. London. https://pubs.aeaweb.org/doi/pdf/10.1257/jep.32.4.227. Accessed 6 Aug 2022

[CR205] Romer PM (1994) The origins of endogenous growth. J Econ Perspect 8(1):3–22

[CR82] Rousseau PL, Wachtel P (2011). What is happening to the impact of financial deeping on economic growth?. Econ Inquiry.

[CR83] Sasongko G, Huruta BE, Huruta AD (2020). Female labor force participation rate in Indonesia: an empirical evidence from panel data approach. Manag Econ Rev.

[CR208] Saqib N (2015) Review of literature on finance-growth Nexus. J Appl Financ Banking 5(4):175–195

[CR210] Schumpeter JA (1911) Theorie der Wirtschaftlichen Entwicklung. Duncker & Humblot, Leipzig

[CR84] Schumpeter JA (2017). Theory of economic development: an inquiry into profits, capital, credit, interest, and the business cycle.

[CR201] Segura AT (2019) A long-run relationship between investment and saving: revisting the Feldstein-Horioka puzzle. Univeristat Jaume I. Available at: http://repositori.uji.es/xmlui/bitstream/handle/10234/186573/TFG_2019_Trilles_Segura_Andrea_.pdf?sequence=1&isAllowed=y

[CR85] Shah PJ (1973) Money and capital in economic development. In: The Collapse of Development Planning-Google Books. Washington, DC: The Brookings Institution

[CR300] Shahbaz M, Bhattacharya M, Mahalik MK (2018) Financial development, industrialization, the role of institutions and government: a comparative analysis between India and China. Appl Econ 50(17):1952–1977

[CR86] Śledziewska K, Akhvlediani T (2017). What determines export performances in high-tech industries. Central Eur Econ J.

[CR87] Smith A (1776) The wealth of nations. In: An inquiry into the nature and causes of the wealth of nation. London, pp 12–15. https://www.birdvilleschools.net/cms/lib/TX01000797/Centricity/Domain/6027/Adam%20Smith%20Wealth%20of%20Nations%20Excerpt.pdf. Accessed 6 Aug 2022

[CR88] Solow RM (1956). A contribution to the theory of economic growth. Q J Econ.

[CR89] Swamy PAVB (1970). Efficient inference in a random coefficient regression model. Econometrica.

[CR91] UNCTAD (2021) Trade and development report 2021: Grom recovery to resilience: the development dimension, United Nations Confe. Geneva. https://unctad.org/system/files/official-document/tdr2021_en.pdf. Accessed 6 Aug 2022

[CR92] United Nations Development Programme (2020a) Labour and employment, Arab development portal. https://www.arabdevelopmentportal.com/indicator/labor-and-employment#:~:text=Labor force participation rate in the region is the lowest,%2C and Syria (41.7%25). Accessed 23 Mar 2022

[CR93] United Nations Development Programme (2020b) Trade, Arab developmental portal. https://data.arabdevelopmentportal.com/. Accessed 24 Mar 2020

[CR94] Valente R (2016). Mainstream and heterodox sources of endogenous growth: some linkages and the role of income distribution. Nierówności Społeczne a Wzrost Gospodarczy.

[CR95] Wang Q, Su M (2020). A preliminary assessment of the impact of COVID-19 on environment–a case study of China. Sci Total Environ.

[CR96] Wang Q, Zhang F (2021). What does the China’s economic recovery after COVID-19 pandemic mean for the economic growth and energy consumption of other countries?. J Clean Prod.

[CR97] Westerlund J, Edgerton DL (2008). A simple test for cointegration in dependent panels with structural breaks. Oxford Bull Econ Stat.

[CR98] Williams K (2018). Has the finance–growth link been broken? Panel data evidence from Latin America and the Caribbean. Economia.

[CR99] World Bank (2021) Middle East and North Africa: recent Development

[CR100] World Bank (2022a) The role of trade in ending poverty, World Bank. https://www.worldbank.org/en/topic/trade/publication/the-role-of-trade-in-ending-poverty. Accessed 6 Aug 2022a

[CR101] World Bank (2022b) Total labor force participation rate, International Labour Organization. https://data.worldbank.org/indicator/SL.TLF.CACT.ZS. Accessed 23 Mar 2022b

[CR102] Zhang H, Kou G, Peng Y (2019). Soft consensus cost models for group decision making and economic interpretations. Eur J Oper Res.

